# A Randomized Comparison Simulating Face to Face Endotracheal Intubation of Pentax Airway Scope, C-MAC Video Laryngoscope, Glidescope Video Laryngoscope, and Macintosh Laryngoscope

**DOI:** 10.1155/2015/961782

**Published:** 2015-06-16

**Authors:** Hyun Young Choi, Young Min Oh, Gu Hyun Kang, Hyunggoo Kang, Yong Soo Jang, Wonhee Kim, Euichung Kim, Young Soon Cho, Hyukjoong Choi, Hyunjong Kim, Gyoung Yong Kim

**Affiliations:** ^1^Department of Emergency Medicine, College of Medicine, Kangnam Sacred Heart Hospital, Hallym University, Seoul 150-950, Republic of Korea; ^2^Department of Emergency Medicine, College of Medicine, Uijeongbu St. Mary's hospital, The Catholic University of Korea, Uijeongbu 480-717, Republic of Korea; ^3^Department of Emergency Medicine, College of Medicine, Hanyang University, Seoul 133-791, Republic of Korea; ^4^Department of Emergency Medicine, CHA University School of Medicine, Seongnam-si, Gyeonggi-do 463-712, Republic of Korea; ^5^Department of Emergency Medicine, Soonchunhyang University Bucheon Hospital, Bucheon 420-767, Republic of Korea; ^6^Department of Emergency Medicine, Ilsan Paik Hospital, Inje University, Ilsan 411-706, Republic of Korea; ^7^Gyeonggi Fire Service Academy, Yongin 449-882, Republic of Korea

## Abstract

*Objectives*. Early airway management is very important for severely ill patients. This study aimed to investigate the efficacy of face to face intubation in four different types of laryngoscopes (Macintosh laryngoscope, Pentax airway scope (AWS), Glidescope video laryngoscope (GVL), and C-MAC video laryngoscope (C-MAC)). *Method*. Ninety-five nurses and emergency medical technicians were trained to use the AWS, C-MAC, GVL and Macintosh laryngoscope with standard airway trainer manikin and face to face intubation. We compared VCET (vocal cord exposure time), tube pass time, 1st ventilation time, VCET to tube pass time, tube pass time to 1st ventilation time, and POGO (percentage of glottis opening) score. In addition, we compared success rate according to the number of attempts and complications. *Result*. VCET was similar among all laryngoscopes and POGO score was higher in AWS. AWS and Macintosh blade were faster than GVL and C-MAC in total intubation time. Face to face intubation success rate was lower in GVL than other laryngoscopes. *Conclusion*. AWS and Macintosh were favorable laryngoscopes in face to face intubation. GVL had disadvantage performing face to face intubation.

## 1. Introduction

Intubation is one of the most important procedures attributing prognosis in severely ill patients [1]. Endotracheal intubation success rates are variable depending on airway structure, patient's clinical status, practitioner's skills, and so forth [2, 3]. The video laryngoscopes, recently and widely used, are good substitutes for conventional direct laryngoscope in difficult airway management [4]. They mount camera lens at the tip of laryngoscope and more curved blade, so that intubation can be performed safely and comfortably with clear and wide internal field of vision [5]. Many emergency physicians are concerned about the feasibility of urgent airway management in limited space in means of transporting patients such as ambulances or helicopters in cases of traffic delays, patients' rapid deterioration of mental state, or entrapped trauma casualties [6]. In prehospital environment in which patients are on the ground or entrapped in vehicles, it is difficult to perform conventional intubation [7–9]. For decades, conventional tracheal intubation was performed at upper side of patient's head. However, mostly, in entrapped patients with restricted position, there is not enough space on patient's head side for tracheal intubation [1]. For this situation, we can try face to face intubation; in other words, inverse intubation can be performed with the provider's face at the same level as the patient's face. There is no needed for another space on patient's head side for tracheal intubation in face to face intubation. Therefore, it can be a very useful method for performing tracheal intubation in restricted position [10]. But it is different from conventional tracheal intubation in the position in which the glottis is viewed and the manipulation of the tube due to the reversely progressing direction. Similarly, since face to face intubation with video laryngoscope differs from conventional intubation, untrained practitioner may feel difficulty performing it.

As described above, face to face intubation will be a good substitute for conventional endotracheal intubation for patients who need urgent endotracheal intubation immediately in limited space [11].

In this study, after teaching the face to face intubation using conventional laryngoscope and video laryngoscopes in manikin model, we analyzed the success rate, time spent, and complications caused by intubation procedure.

## 2. Methods

The Institutional Review Board at Hallym University Kangnam Sacred Heart Hospital approved this study. IRB number was 2014-11-153.

### 2.1. Subject

Ninety-five nurses and emergency medical technicians (EMT) participated in a 2-day long airway management education program in Gyeonggi-do fire service academy, South Korea. They were divided into 4 groups and each group was trained in consecutive order.

### 2.2. Study Design

Instructors gave lectures during 2 hours about endotracheal intubation and airway managements. The lecture session was followed by practice session. They were divided into 4 groups. Each group took 4 different 50-minute long practices which include endotracheal intubation using Macintosh blade and video laryngoscopes and face to face intubation. It took 4 hours in total.

After the practice session, the subjects were divided into four groups and each group took checklist for the test for tracheal intubation. Four groups were divided by kinds of laryngoscopes, direct laryngoscope (Macintosh blade, #4), Pentax airway scope (AWS, Hoya, Tokyo, Japan), C-MAC video laryngoscope pocket with standard #3 blade model (C-MAC, Karl Storz Endoscopy, Tuttlingen, Germany), and Glidescope video laryngoscope with standard #3 blade model (GVL, Verathon Medical Inc., Bothell, WA). And we used Laerdal airway management trainer (Laerdal, Medical Corporation, Stavanger, Norway) which is as widely used manikin for training of airway management.

Instructors checked and recorded POGO (percentage of glottis opening) and times when glottis was visible and when endotracheal tube passed vocal cord. They also checked chest rising of manikin, which was recorded as 1st ventilation, using tube ballooning and ventilation with bag-valve mask.

All the tests were performed in separated space. Before test, every subject received random test sequence table. Test sequence of laryngoscope types were determined by random sample.

### 2.3. Statistical Analysis

Statistical analysis was carried out with the 22.0 version of the SPSS program for windows (SPSS Inc., Chicago, IL, USA). Data was presented as mean ± standard deviation (SD). In previous study, total intubation time for face to face intubation was 21.6 ± 10.1 seconds [10]. To detect 20% difference in total intubation time with a power of 0.9 and *α* = 0.05, we estimate that 75 subjects would be adequate considering a 20% drop rate. We used Shapiro-Wilk test for verifying normal distribution and Wilcoxon signed rank test for verifying the result which is not according to normal distribution. A significant difference was considered when *P* value was less than 0.05. For comparison in correlation of multiple variables, we used Friedman test and applied Bonferroni's method for Post hoc analysis.

## 3. Result

95 subjects participated in this study but we excluded 9 subjects due to informational errors such as missing data on evaluation form. So, 86 subjects were enrolled in this study. They consisted of 54 men (62.8%) and 32 women (37.2%) and were classified into 17 nurses (19.8%), 68 1st level EMT (emergency medical technicians) (79.0%), and 1 2nd level EMT (1.2%). 1st level EMT was licensed to college graduates of emergency medical technology; otherwise, 2nd level EMT was licensed by passing written and practical test for emergency situations. In South Korea, most of healthcare providers in the field consisted of 1st level EMT, 2nd level EMT, and nurses. Mean age of subjects was 28.3 years old; mean career as healthcare provider was 3.6 years. Most of them (83 of 86) experienced intubation less than 3 times. In addition, they never experienced intubation using video laryngoscopes and face to face intubation (Table 1). We described the result divided into VCET (vocal cord exposure time), POGO (percentage of glottis opening) score, tube pass time and 1st ventilation time. In addition, we calculated spent time from VCET to tube pass time and from tube pass time to 1st ventilation time. We limited subject's data in case of successful endotracheal intubation achieved only in 1st attempt. We compared the success rate with the number of attempts and regarded a failure in case of not achieving endotracheal intubation within 1 minute because we assumed the emergency situation in which subjects must achieve face to face intubation in spite of very narrow space; in other words, they cannot wait for conventional intubation and needed more space [6, 12]. Finally, we described complication by kinds of laryngoscope.

### 3.1. VCET

Vocal cord exposure time (VCET) means the time taken by the subject to hold a handle of laryngoscope to find a vocal cord opening. In Macintosh blade, the VCET was 7.8 ± 3.3 seconds and it was faster than other video laryngoscopes, in AWS 10.9 ± 7.8, in GVL 8.4 ± 4.9, and in C-MAC 8.4 ± 4.6. But, no significant difference showed among laryngoscopes (*P* = 0.199 in Friedman test) (Table 2).

### 3.2. POGO Score

Percentage of glottis opening (POGO) score defined a certain extent of visualized vocal cord after insertion of laryngoscope to oral cavity. If we can watch whole vocal cord, we mark it 100%, and if we cannot find vocal cord, we mark it 0%. In Macintosh blade (53.6 ± 22.3%), POGO score was lower than all kinds of laryngoscopes that we used and showed significant difference. Compared to Macintosh blade, POGO score in AWS was 81.7 ± 18.3% (*P* = 0.000), in GVL was 65.4 ± 25.0% (*P* = 0.000), and in C-MAC was 72.9 ± 20.8% (*P* = 0.000). In comparison of video laryngoscopes, AWS showed higher POGO score than GVL (*P* = 0.000) and C-MAC (*P* = 0.002), but there was no significant difference between GVL and C-MAC (*P* = 0.188) (Tables 2 and 3).

### 3.3. Tube Pass Time

Tube pass time means time spent by subjects to grasp a handle of blade passing the vocal cord by endotracheal tube. Macintosh blade (18.7 ± 7.3 sec) was faster than GVL (26.8 ± 10.0 sec, *P* = 0.001) and C-MAC (22.8 ± 10.2 sec, *P* = 0.000) and showed no difference with AWS (19.6 ± 9.5 sec, *P* = 0.608). In comparison among video laryngoscopes, AWS was faster than GVL (*P* = 0.005) but there were no differences between AWS and C-MAC and GVL and C-MAC (*P* = 0.011, *P* = 0.108) (Tables 2 and 3).

### 3.4. 1st Ventilation Time

1st ventilation time means time spent from holding a handle of laryngoscope and passing the vocal cord by endotracheal tube to give 1st ventilation via inserted endotracheal tube. It also means total intubation time. There were no differences between Macintosh blade (28.4 ± 7.7 sec) and AWS (29.6 ± 10.9 sec) and GVL (39.2 ± 9.7 sec) and C-MAC (35.2 ± 10.4 sec) (*P* = 0.530, *P* = 0.207). However, Macintosh blade was faster than GVL (*P* = 0.000) and C-MAC (*P* = 0.000); AWS was also faster than GVL (*P* = 0.003) and C-MAC (*P* = 0.000) (Tables 2 and 3).

### 3.5. VCET to Time of Endotracheal Tube Passing Vocal Cord (Tube Pass Time)

It means time spent from vocal cord exposure to passing the endotracheal tube. Macintosh blade (10.9 ± 5.9) and AWS (10.4 ± 10.9) showed similar result, and there is no significant difference (*P* = 0.028). In GVL (22.8 ± 27.1) and C-MAC (17.1 ± 14.3), they needed more time from vocal cord exposure to endotracheal tube passing via vocal cord compared to Macintosh blade and AWS, individually (Macintosh versus GVL, *P* = 0.003; Macintosh versus C-MAC, *P* = 0.001; AWS versus GVL, *P* = 0.001; AWS versus C-MAC, *P* = 0.000). No significant difference showed between GVL and C-MAC (*P* = 0.161) (Tables 2 and 3).

### 3.6. Time of Endotracheal Tube Passing Vocal Cord (Tube Pass Time) to 1st Ventilation Time

9.6 ± 3.9 seconds are needed from tube passing to 1st ventilation in Macintosh blade, and that, in AWS, was 9.8 ± 3.7 seconds. In GVL and C-MAC, the results were 10.6 ± 9.7 seconds and 12.2 ± 4.5 seconds. In comparison with Macintosh blade, AWS (*P* = 0.860) and GVL (*P* = 0.060) did not show significant difference, but C-MAC (*P* = 0.000) was meaningfully slower than Macintosh blade. AWS was faster than C-MAC with significant difference (*P* = 0.000), but there was no difference with GVL (*P* = 0.171). No significant difference was found between GVL and C-MAC (*P* = 0.165) (Tables 2 and 3).

### 3.7. Endotracheal Intubation Success Rate according to the Number of Attempts

**Figure 1:**
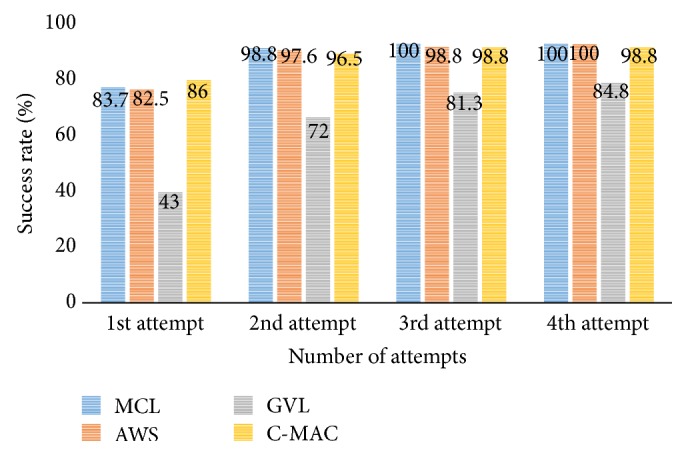
Endotracheal intubation success rate according to the number of attempts. Glidescope video laryngoscope (GVL) showed lower success rate compared with other laryngoscopes in all of attempts. MCL: Macintosh laryngoscope; AWS: Pentax airway scope; GVL: Glidescope video laryngoscope; C-MAC: C-MAC video laryngoscope.

86 subjects performed face to face intubation using laryngoscopes; we determined a failure in which subjects did not achieve endotracheal intubation within 1 minute or accomplished esophageal intubation. In Macintosh blade, 72 subjects (83.7%) achieved successful endotracheal intubation in first attempt and 85 (98.8%) succeeded in second attempt. At third attempt, all subject (100%) succeeded in endotracheal intubation. 71 subjects (82.5%) succeeded in endotracheal intubation in first attempt using AWS. In second attempt, 84 (97.6%) achieved successful endotracheal intubation; 85 (98.8%) succeeded in third attempt; all subject (100%) succeeded in fourth attempt. In GVL, 37 subjects (43.0%) succeeded in endotracheal intubation at first attempt, and 62 (72.0%) succeeded in second attempt. 70 subjects (81.3%) succeeded in third attempt, and 73 (84.8%) in the fourth. 13 subjects (15.2%) did not achieve successful intubation during four attempts. 74 subjects (86.0%) succeeded in endotracheal intubation in first attempt using GVL, 83 subjects (96.5%) in second attempt, and 85 (98.8%) in third attempt. 1 subject (1.2%) failed in endotracheal intubation during four attempts (Figure 1, Table 4).

### 3.8. Complication

We examined complications of face to face intubation during procedure for tooth injury and esophageal intubation. We included all attempts of endotracheal intubation. Tooth injury was considered when tester heard tooth click. The number of tooth injuries in Macintosh blade was one, in AWS and GVL five, and, else, in C-MAC three. One esophageal intubation occurred in Macintosh blade, and no esophageal intubation occurred in video laryngoscopes.

## 4. Discussion

Endotracheal intubation is very important procedure in emergency department for severely ill patients [13, 14]. In conventional intubation, operator sets a location at the upper side of patient's head, grasps a laryngoscope with left hand, and inserts endotracheal tube by right hand. For better visualization, operator shift patient's tongue to left using blade. Lots of new techniques and machines are introduced for easy successful intubation, but, until recently, conventional intubation is the most commonly used method for airway management. Video laryngoscopes, for difficult airway, consist of a laryngoscope with a light source and a camera in distal blade. In contrast to conventional blade having about 15′-visual field, video laryngoscopes make wider visual angle because of the camera on distal blade [4]. In special situation, such as, in ambulance or helicopter, occasionally, there is no space at patient's upper side for conventional intubation, So operator has to intubate on lateral or frontal side of patient [9]. In contrast to conventional laryngoscopy, the practitioner holds the handle of the laryngoscope in his right hand with the top of the blade in the upright position. After the opening of patient's airways, the top of the laryngoscope's curved blade will be in place in the left part of the patient's mouth [10].

We examined this study to investigate whether video laryngoscopes are helpful in special situation like being entrapped in car or permitted narrow space and whether video laryngoscopes are more useful than conventional intubation set (Macintosh blade). We choose several video laryngoscopes: AWS which has endotracheal tube-guiding groove channel in distal blade (P blade), GVL with difficult blade for difficult airway that has elliptically tapered blade shape rising to distal, and C-MAC with conventional blade which has the same blade angle compared with Macintosh blade [4].

We discussed the course of face to face intubation categorized as VCET, POGO score, VCET to tube pass time, tube pass time to 1st ventilation time, success rate by number of attempts and complications.

### 4.1. Face to Face Intubation versus Supraglottic Airway Devices Insertion

In previous simulation study, supraglottic airway devices (SAD) are faster than Macintosh laryngoscope in entrapped situation [15]. Hence, SAD insertion can be considered to be more useful method compared with endotracheal intubation. However, in cases of lung injuries or massive bleeding or vomitus in oral cavity, SAD alone is not enough to secure airway. In these cases, endotracheal intubation is preferred to SAD insertion for providing high oxygen concentration [16].

In case of severe injury with restricted position, face to face intubation is a reasonable choice.

### 4.2. Vocal Cord Exposure Time (VCET)

No significant difference was detected among all laryngoscopes (*P* = 0.199). Macintosh blade had advantage of easy insertion to oral cavity because it had smaller blade than other video laryngoscopes due to its simplicity; however, video laryngoscopes had camera at the mount of blade tip, so subjects easily detected vocal cord so long as blade of video laryngoscope was inserted in oral cavity.

### 4.3. POGO (Percentage of Glottis Opening) Score

Macintosh blade showed lower POGO score than all laryngoscopes, in AWS (*P* = 0.000) and GVL (*P* = 0.000) and C-MAC (*P* = 0.000). In comparison among video laryngoscopes, AWS showed higher POGO than GVL (*P* = 0.000) and C-MAC (*P* = 0.002). GVL with C-MAC did not show significant difference (*P* = 0.016, *P* = 0.022). Video laryngoscopes were made for difficult airway management and gave us advanced vision compared to Macintosh blade [17]. In case of face to face intubation, similar to conventional intubation, the visual field is wider and POGO is higher in video laryngoscopes than Macintosh blade. GVL with difficult blade had more curved angle than other blades, so it was difficult to expose vocal cord in face to face intubation performing on opposite side of conventional intubation.

### 4.4. VCET to Tube Pass Time

VCET to tube pass time means spending time from vocal cord exposure to pass it. Between Macintosh blade and AWS, there was no significant difference (*P* = 0.028). Macintosh was faster than GVL (*P* = 0.003) and C-MAC (*P* = 0.001). Among video laryngoscopes, AWS was faster than GVL and C-MAC (*P* = 0.001, *P* = 0.000). There was no significant difference between GVL and C-MAC (*P* = 0.161).

It may be due to eye-hand discordance. In case of face to face intubation using Macintosh blade, operator sets location on upper side of patient's head, checks vocal cord with the naked eye, and inserts endotracheal tube to vocal cord. On the other hand, operator with video laryngoscopes will be watching monitor showing view from end of video laryngoscope which is in contrast angle to hand direction [18]. Operator inserts endotracheal tube to vocal cord watching screen attached to video laryngoscopes, but the direction of manipulating endotracheal tube and the location of vocal cord on screen to advanced direction of endotracheal tube is different [19, 20]. So, it is difficult to insert endotracheal tube quickly and precisely. Difficult blade of GVL has larger angle of blade for difficult airway compared to conventional blade, so it is more difficult to manipulate endotracheal tube due to more distorted up-and-down angle.

### 4.5. Tube Pass Time to 1st Ventilation Time

Macintosh was faster than C-MAC (*P* = 0.000) and showed no significant difference with AWS (*P* = 0.860) and with GVL (*P* = 0.060). In comparison among video laryngoscopes, AWS was faster than C-MAC (*P* = 0.000). AWS and GVL and GVL and C-MAC did not show significant difference (*P* = 0.171, *P* = 0.165).

We did not know the definite cause of why C-MAC was slower than Macintosh blade and AWS. Maybe, in face to face intubation, the monitor attached to C-MAC was inversely rotated. Most operators in this study tried rotating C-MAC monitor to its original positon taking up more time. We thought it requires further investigation about other causes.

### 4.6. Success Rate according to the Number of Attempts

In Macintosh blade, AWS, and C-MAC, they showed over 80% success rate in first attempt (Table 4). Otherwise, only 43% of subjects achieved successful intubation in GVL. For second attempt, 72% of subjects succeeded in GVL; the others showed over 95% success rate. After fourth trial, Macintosh and AWS showed 100% success rate; C-MAC showed 98.8%. But GVL showed only 84.8% of success rate, and 15.2% of subjects could not achieve successful face to face intubation during four times of attempts (Figure 1). GVL had difficult blade for difficult airway; in contrast to other laryngoscopes, eye-hand discordance was more severe.

### 4.7. Complication

One tooth injury occurred in Macintosh, five injuries in AWS, five injuries in GVL, and three injuries in C-MAC. AWS had bigger and thicker blade than others, it contributed to tooth injury. In performing intubation using GVL, as appeared by the result in which GVL showed lower successful face to face intubation rate, subjects seemed to move more in oral cavity than other laryngoscopes for successful intubation; we guessed it influenced broken tooth. Esophageal intubation occurred once only in Macintosh blade; it seemed to be meaningless.

### 4.8. Limitation

First, we cannot exclude learning effect of laryngoscopes. Though subjects performed face to face intubation using multiple laryngoscopes via randomized serial, subjects might be trained four times of serial face to face intubation and perform better as time goes by. Second, it is simulation study using manikin, not a patient. In this study, all manikins lay down on floor, not in sitting position. Face to face intubation is very useful in patient of entrapped car, mostly sitting. In addition, there is enough space at upper manikin's head side to perform face to face intubation compared to narrow space such as ambulance and helicopter.

It is not unusual that operator suffers poor visual field due to secretion or blood or vomitus on performing CPR in field. Sometimes, intubation was delayed for cleaning lens of video laryngoscopes. However, in case of Macintosh blade, securing visual field was faster because direct suction was possible in insertion state of Macintosh blade. So the result could not adapt to patients exactly. Third, subjects had no experience of face to face intubation, but they took lots of lectures about airway management using manikin. So they have familiarity of intubation using manikin study compared to someone who has no prior education.

Fourth, we simulated this study and assumed an emergency situation in which the victim needed emergent face to face intubation in limited study; we determined that the failure time of intubation was 1 minute. We thought it might be a cause of low success rate in first intubation attempt using GVL. We compared spent time only in success cases at first time attempt of all kinds of laryngoscopes. So, we guessed that GVL took a long time for face to face intubation compared to our result. Next study, if that is possible, it will give the opportunity for successful intubation without limited time and it will be more correct in comparison to intubation time among laryngoscopes.

## 5. Conclusion

In limited space and restricted position with emergent situation, face to face intubation is a useful substitute for conventional intubation. However, its success rate is different due to multiple causes, for example, eye-hand discordance. Macintosh blade and AWS showed significantly faster intubation time than GVL and C-MAC, in face to face intubation.

## Figures and Tables

**Table 1:** Demographic characteristics of subjects (*n* = 86).

Characteristics	Data
Sex (*n*, percent)	Male (54, 62.8%)
Age (years, range)	28.3 (21–40)
Work experience as healthcare provider (years)	3.6
0 (*n*, percent)	27 (31.4%)
to 5 years	36 (41.9%)
>5 years	23 (26.7%)
License	
Nurse	17 (19.8%)
1st level EMT	67 (77.9%)
2nd level EMT	1 (1.2%)
Intubation experience	
MCL <3 (times)	83 (96.5%)
Video laryngoscope <3 (times)	86 (100%)
Face to face intubation <1	86 (100%)

EMT: emergency medical technician; MCL: Macintosh laryngoscope.

**Table 2:** Comparison of intubation time (sec) and POGO (%) according to laryngoscopes (mean ± SD).

	MCL	AWS	GVL	C-MAC	*P* value
VCET (sec)	7.8 ± 3.3	10.9 ± 7.8	8.4 ± 4.9	8.4 ± 4.6	0.199
POGO (%)	53.6 ± 22.3	81.7 ± 18.3	65.4 ± 25.0	72.9 ± 20.8	0.000
Tube pass time (sec)	18.7 ± 7.3	19.6 ± 9.5	26.8 ± 10.0	22.8 ± 10.2	0.001
1st ventilation time (sec)	28.4 ± 7.7	29.6 ± 10.9	39.2 ± 9.7	35.2 ± 10.4	0.000
VCET to tube pass time (sec)	10.9 ± 5.9	10.4 ± 10.9	22.8 ± 27.1	17.1 ± 14.3	0.000
Tube pass time to 1st ventilation time (sec)	9.6 ± 3.9	9.8 ± 3.7	10.6 ± 9.7	12.2 ± 4.5	0.000

^*∗*^ *P* value < 0.05 is level of statistical significance according to Friedman test.

MCL: Macintosh laryngoscope; AWS: Pentax airway scope; GVL: Glidescope video laryngoscope; C-MAC: C-MAC video laryngoscope.

**Table 3:** Statistical significance (*P* value) among laryngoscopes for intubation time (sec) and POGO (%).

	MCL versus AWS (*P* value)	MCL versus GVL (*P* value)	MCL versus C-MAC (*P* value)	AWS versus GVL (*P* value)	AWS versus C-MAC (*P* value)	GVL versus C-MAC (*P* value)
VCET	0.008	0.038	0.217	0.756	0.090	0.220
POGO	0.000^∗^	0.000^∗^	0.000^∗^	0.000^∗^	0.002^∗^	0.188
Tube pass time	0.608	0.001^∗^	0.000^∗^	0.005^∗^	0.011	0.108
1st ventilation time	0.530	0.000^∗^	0.000^∗^	0.003^∗^	0.000^∗^	0.207
VCET to tube pass time	0.028	0.003^∗^	0.001^∗^	0.001^∗^	0.000^∗^	0.161
Tube pass time to 1st ventilation time	0.860	0.060	0.000^∗^	0.171	0.000^∗^	0.165

^*∗*^ *P* value < 0.008 is level of statistical significance according to Bonferroni's method.

MCL: Macintosh laryngoscope; AWS: Pentax airway scope; GVL: Glidescope video laryngoscope; C-MAC: C-MAC video laryngoscope.

**Table 4:** Success rate according to the number of attempts.

	MCL	AWS	GVL	CMAC
Success at 1st attempt	72 (83.7%)	71 (82.5%)	37 (43.0%)	74 (86.0%)
Success at 2nd attempt	13 (15.1%) (85, 98.8%)	13 (15.1%) (84, 97.6%)	25 (29.0%) (62, 72.0%)	9 (10.4%) (83, 96.5%)
Success at 3rd attempt	1 (1.1%) (86, 100%)	1 (1.1%) (85, 98.8%)	8 (9.3%) (70, 81.3%)	2 (2.3%) (85, 98.8%)
Success at 4th attempt	0	1 (1.1%) (86, 100%)	3 (3.4%) (73, 84.8%)	0

Failure at 4th attempt	0	0	13 (15.1%)	1 (1.1%)

MCL: Macintosh laryngoscope; AWS: Pentax airway scope; GVL: Glidescope video laryngoscope; C-MAC: C-MAC video laryngoscope.
